# Uremic Tumoral Calcinosis of the Cervical Spine With Symptomatic Myelopathy: An Illustrative Case

**DOI:** 10.7759/cureus.110439

**Published:** 2026-06-08

**Authors:** Aashka Sheth, Nicholas Dietz, Andrea Becerril-Gaitan, Edward Ham, Sahar Almustafa, Emily Sieg

**Affiliations:** 1 Neurosurgery, University of Louisville School of Medicine, Louisville, USA; 2 Neurosurgery, University of Louisville Hospital, Louisville, USA; 3 Pathology, University of Louisville Hospital, Louisville, USA

**Keywords:** case report, chronic kidney disease, myelopathy, spine, tumoral calcinosis

## Abstract

Tumoral calcinosis is a rare pathology characterized by extraarticular soft tissue calcium deposition, commonly near large joints. Tumoral calcinosis may be idiopathic or associated with underlying disorders, such as end-stage renal disease (ESRD), sarcoidosis, and hyperparathyroidism. The cervical spine is a rare location for tumoral calcinosis, with only 11 cases of uremic tumoral calcinosis previously reported in the literature. We report a case of cervical tumoral calcinosis in a 46-year-old man with end-stage renal disease on peritoneal dialysis. He presented with unilateral extremity weakness and was found to have a C2-C6 soft tissue calcific mass. His hospital course was complicated by medical instability, and he eventually underwent mass resection, laminectomy, and posterior spinal fusion (PSF). After surveying the literature, we found that 36 cases of cervical tumoral calcinosis have previously been reported. Laminectomy and further medical management are reasonable treatment strategies to alleviate symptoms and prevent recurrence. Patients with ESRD represent a high-risk operative population, with increased intra- and postoperative complications. Although tumoral calcinosis rarely occurs in the spine, it is an important condition for spinal surgeons to be aware of, as tumoral calcinosis may be a more frequently encountered pathology due to the rising prevalence of chronic kidney disease and the aging population.

## Introduction

Tumoral calcinosis is a rare, benign extraosseous pathology characterized by calcium deposition most commonly reported near large joints such as the hip, shoulder, and elbow [1,2]. Typical symptoms involve pain, swelling, and the functional impairment of the affected joint [3], whereas spinal involvement is rarely seen [1,4]. Tumoral calcinosis may be considered a complication of renal failure as it is seen in 1.6% of cases of end-stage renal disease (ESRD) [5], and it can also be associated with hyperparathyroidism, sarcoidosis, and scleroderma [1,2]. Management is complicated by diagnostic challenges due to a lack of clear clinical symptoms and characteristic imaging findings, resulting in frequent misdiagnosis [1]. There is no established standard treatment, and management is usually dependent on size, location, and relation to other structures, but surgical excision can be beneficial in treating early stages of tumoral calcinosis [6].

Spinal tumoral calcinosis is especially uncommon, with only 7% of reported tumoral calcinosis cases occurring in the spine [7]. Tumoral calcinosis at this level typically presents with symptoms of cervical myelopathy or pain, with imaging showing nonspecific mass-like lesions that may resemble neoplasms [8]. Only 11 cases of uremic tumoral calcinosis have been reported in the cervical spine within the PubMed database. We report a case of spinal tumoral calcinosis in a medically complex patient with ESRD.

## Case presentation

In November 2025, a 46-year-old man with a history of ESRD secondary to uncontrolled diabetes mellitus presented to the emergency department after one day of acute left upper and lower extremity motor weakness. Physical examination revealed a profound weakness of the left upper and lower extremity, with unaffected sensation, and a palpable neck mass. Hoffman's sign was positive in the left extremity. Admission laboratory studies are depicted in Table 1. Based on his physical examination and visible neck mass, imaging was ordered due to a high suspicion of a tumor causing mass effect.

**Table 1:** Patient's laboratory results on admission.

Parameters	Laboratory Results on Admission	Reference Range
Calcium	7.2 mg/dL	8.4-10.2 mg/dL
Phosphorous	9.6 mg/dL	3.0-4.5 mg/dL
Glucose	30 mg/dL	70-110 mg/dL
Creatinine	11.3 mg/dL	0.6-1.2 mg/dL
Blood urea nitrogen	51 mg/dL	7-18 mg/dL
White blood cells	28,800 μL	4,500-11,000 μL

Initial computed tomography (CT) showed heterotopic bone formation involving the right acromioclavicular joint and cervical spine (Figure 1, panel C) and a 1.4 × 5.9 × 6.1 cm soft tissue calcific density extending from the coccyx into adjacent soft tissue. CT of the neck delineated the previously seen lobulated calcified mass adjacent to the spinous processes in the cervical spine with well-marginated borders and sclerotic appearance suggestive of tumoral calcinosis (Figure 2). Further workup with magnetic resonance imaging (MRI) of the cervical spine demonstrated a 2.8 × 5.2 × 5.9 cm mass arising from the C6 spinous and extending into the posterior left extradural space at the levels of C2-C6, with imaging characteristics consistent with tumoral calcinosis (Figure 3). This lesion had dependent T2 hypointensities with anti-dependent T2 hyperdensities within this lesion, consistent with the sedimentation sign. Varying degrees of cervical canal stenosis due to the extension of tumoral calcinosis into the extradural space were seen from C3 to C6, most prominent at the C5-C6 level. CT of the chest showed a lobulated calcified soft tissue mass associated with the right scapula and a similar lesion at the left scapula and shoulder.

**Figure 1:**
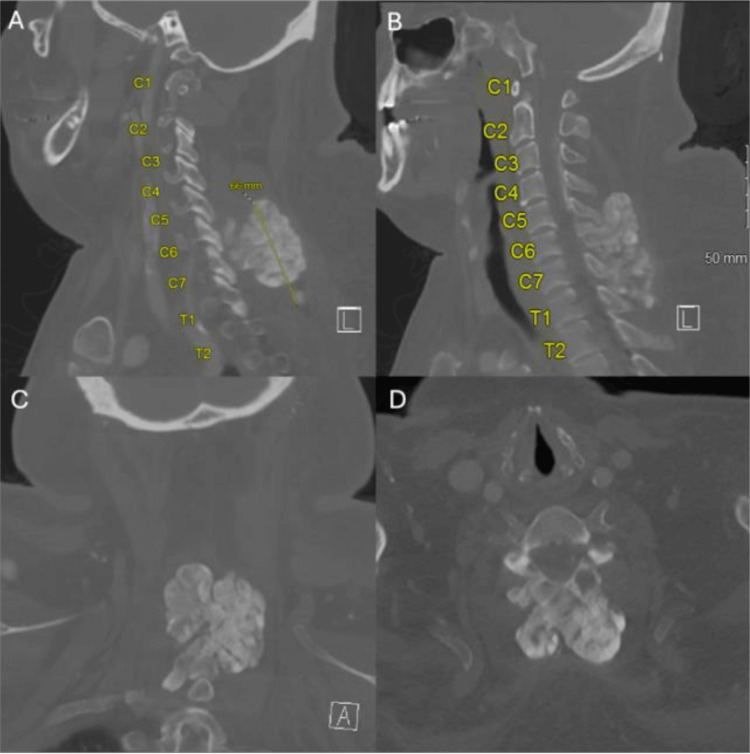
Computed tomography (CT) of neck soft tissue with contrast, bone window. Sagittal CT demonstrating a large, calcified mass in the paraspinous muscles measuring 6.4 × 5.0 × 6.9 cm (panel A). The mass-like lesion is adjacent to spinous processes and extends from C5 to C7 (panel B). Coronal CT shows the well-marginated borders and sclerotic appearance suggestive of tumoral calcinosis (panel C). Axial CT at the level of C5-C6 demonstrates the lesion accompanied by calcifications along the ligamentum flavum that contribute to severe canal stenosis (panel D).

**Figure 2:**
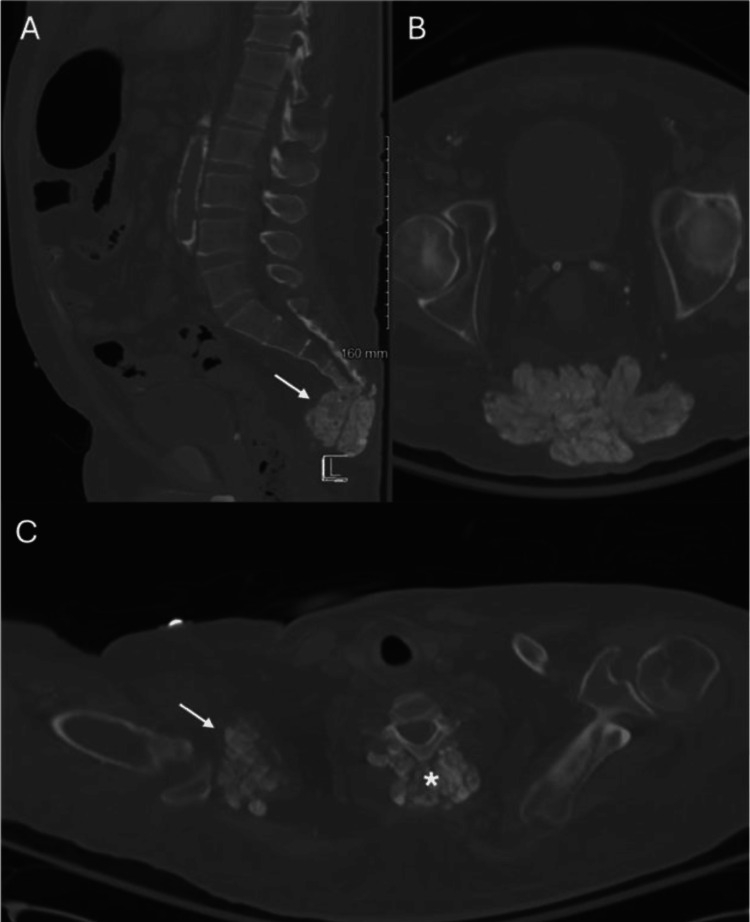
Initial computed tomography (CT) of the chest, abdomen, and pelvis without contrast, bone window. Non-contrast sagittal abdomen/pelvis CT demonstrating a 1.4 × 5.9 × 6.1 cm soft tissue calcific density surrounding the coccyx (white arrow, panel A). Axial (panel B) view showing the large heterotopic calcific density formation exophytic from the coccyx extending into the adjacent soft tissues and bilateral ischial tuberosities. Axial chest CT (panel C) demonstrating sites of heterotopic bone formation at the right acromioclavicular joint (white arrow) and posteriorly at the cervical spine (white asterisk).

**Figure 3:**
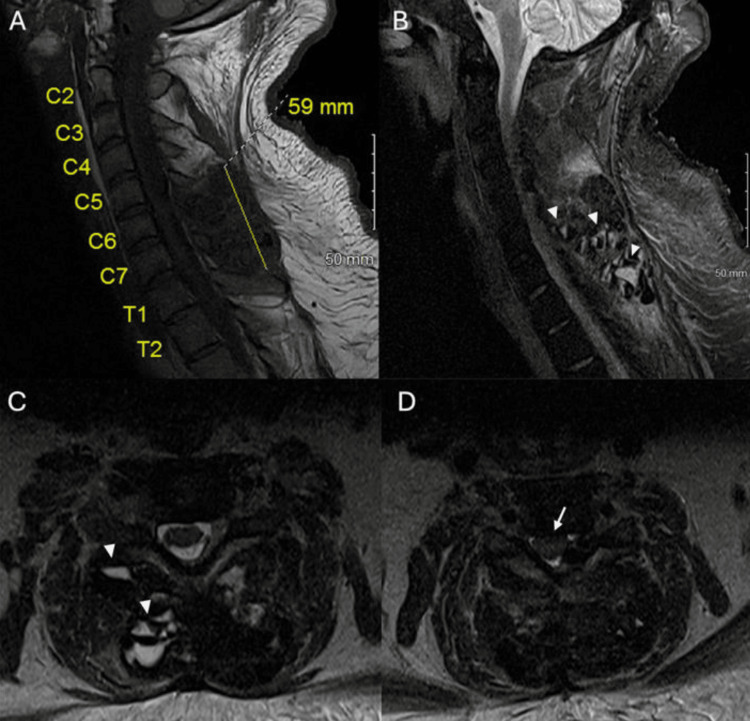
Cervical magnetic resonance imaging (MRI) without contrast. Sagittal T1-weighted sequence (panel A) demonstrating a 2.8 × 5.2 × 5.9 cm mass arising from the C6 spinous process with extension to the posterior soft tissues at the level of C5-C7. Sagittal T2-weighted STIR sequence (panel B) showing increased hyperintensity surrounding the aforementioned mass, which extends into the extradural space from C2 to C6, resulting in varying degrees of spinal canal stenosis. Characteristic sedimentation signs, representing the fluid-calcium levels, are shown in panels B and C (white arrowheads). Axial T2-weighted sequence demonstrating extension into the posterior extradural space resulting in severe cervical stenosis and invasion into the left neural foramina at the C5-C6 level (panel D, white arrow). STIR: short tau inversion recovery

The patient's initial hospital course was complicated by an acute ischemic stroke involving the left caudate on hospital day 4, requiring ICU admission and delaying planned cervical spine surgery. The patient required endotracheal intubation on hospital day 5 due to acute hypoxic respiratory failure. On hospital day 10, once medically stable, the patient was taken to the operating room (OR) for a C3-C6 laminectomy, mass resection, and C2-T2 posterior spinal fusion (PSF). Intraoperatively, electrocautery was used to expose the cervical mass, which was associated with chalky white non-purulent drainage (Figure 4). The mass was resected and sent for pathology. After the resection of the mass, the spinous processes of C2-C5 were exposed. Throughout the procedure, it was noted that the patient had poor bone quality, likely in the setting of his ERSD. After spinal instrumentation was completed, the laminectomy was done, and the epidural portion of the lesion was carefully peeled off the calcified dura; two drains were placed inferiorly, and the wound was closed. The histopathological analysis of the mass revealed nodular to irregular aggregates of amorphous to basophilic calcium deposits surrounded by foreign body giant cell reaction, features consistent with a diagnosis of tumoral calcinosis (Figure 5).

**Figure 4:**
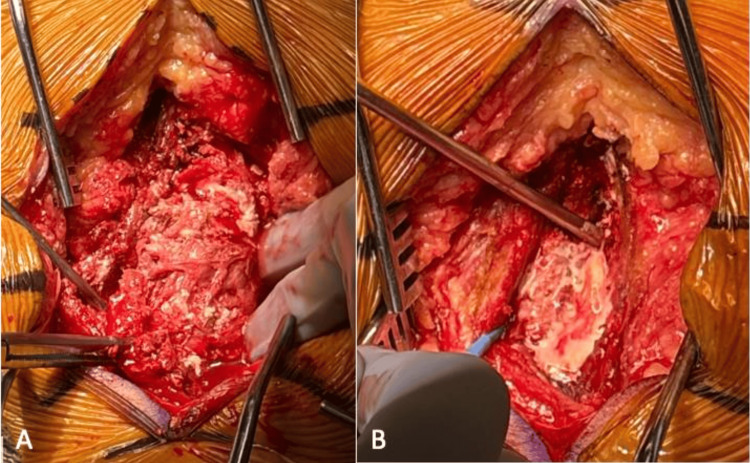
Intraoperative exposure and resection of cervical mass. (A) Exposed calcified C2-C6 cervical mass prior to resection. (B) Chalky white drainage released by the resection of cervical mass, a characteristic of tumoral calcinosis.

**Figure 5:**
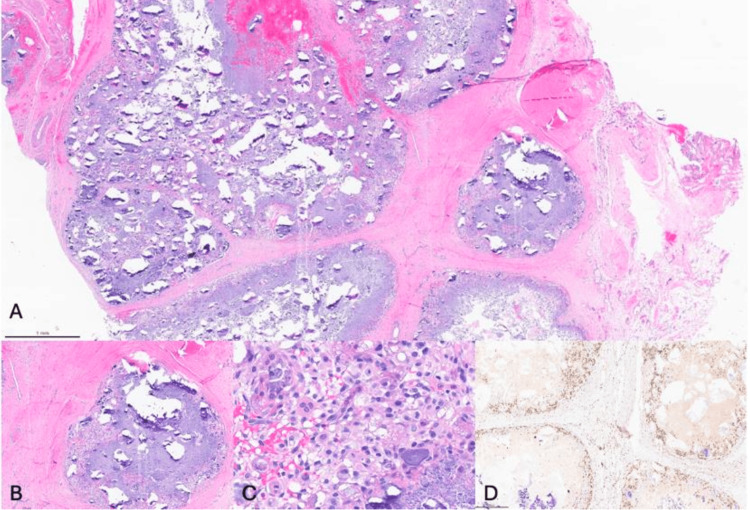
Histopathological specimen demonstrating features consistent with tumoral calcinosis. (A) Low‑magnification (2×) hematoxylin and eosin (H&E)-stained section showing discrete nodules of dystrophic calcification separated by bands of fibrous stroma. (B) Intermediate‑magnification (5×) H&E section further delineating the architecture of a representative calcified nodule. (C) High‑magnification (40×) H&E section showing a prominent histiocytic inflammatory response concentrically arranged around the calcified material. (D) CD163 immunohistochemical stain highlighting the population of CD163‑positive histiocytes surrounding the calcific deposits.

Postoperatively, the patient remained intubated and required multiple vasopressor infusions with concern for hypovolemic shock. Drains were removed on postoperative day 3. The patient had a complicated postoperative course with slow recovery in the setting of his multiple preoperative comorbidities and was discharged to a long-term acute care facility on postoperative day 20.

## Discussion

Tumoral calcinosis is usually characterized by solitary lesions, presenting in patients before the second decade of life [9]. The etiology of tumoral calcinosis is not well-understood, but multiple theories have been proposed. Idiopathic lesions are the most prevalent type, with 30% associated with positive family history [10]. Other explanations include dystrophic calcification caused by injured tissue and secondary tumor calcinosis due to underlying disease such as chronic renal failure, primary or secondary hyperparathyroidism, and scleroderma [6,11]. The proposed mechanism for tumoral calcinosis is an increased production of calcium phosphate, driven by hyperphosphatemia [1,9]. Hyperphosphatemia results in recurrent trauma at the local tissue level, with chronic inflammation leading to calcification and fibrosis surrounding bone tissue [11].

The diagnosis of tumoral calcinosis is mainly based on imaging showing multilobulated and cystic periarticular calcifications [6]. CT can be used as a guide for surgical planning by demonstrating the extent and relations of lesions. Histopathology is used to confirm a diagnosis of tumoral calcinosis, with biopsy demonstrating calcifications with liquid chalklike contents [9].

Treatment strategy is based on the type and stage of the lesion, with consideration of site, size, and surrounding anatomical structures [9]. Surgical excision remains the primary treatment strategy for early stages or symptomatic lesions causing functional impairment [2,4,9]. In the setting of spinal tumoral calcinosis, laminectomy, partial lesion resection, and spinal fusion have been associated with favorable outcomes [2]. As there is a high rate of recurrence, patients may require subsequent monitoring and repeat excisions [4,9]. The treatment of tumoral calcinosis secondary to renal disease or dialysis-related tumoral calcinosis is mainly medical, including calcium- and phosphate-restricted diets, dialysates, and phosphate binders [9].

There have been 36 cases of cervical tumoral calcinosis reported in the literature (Table 2). Scleroderma was the most common etiology, reported in 41.6% of cases [12-24]. Eleven patients had a history of ESRD [25-35]. Eight cases were idiopathic [8,36-42]. One patient had a history of osteoarthritis, and one patient had hyperparathyroidism [39,43]. Tumoral calcinosis has a higher prevalence in women, with only 16.7% of reported cervical tumoral calcinosis cases occurring in men. The age range of patients affected was four months to 90 years. The most common presenting symptom was myelopathy, with pain, numbness, and weakness frequently reported. Most cases were successfully treated via resection and laminectomy. Four patients experienced symptomatic improvement with medical management [14,22,26,32]. Our patient was a 46-year-old man with ESRD who developed a cervical mass, causing left-sided weakness. Surgery was delayed due to medical instability, and his postoperative course was complicated by these comorbidities. Patients with ESRD have significantly increased surgical risks, with higher rates of postoperative myocardial infarction, stroke, sepsis, electrolyte imbalances, and intubation [44]. This is reflected in the current case, as our patient experienced a preoperative ischemic stroke, delaying surgery, and remained intubated postoperatively, with additional concern for myocardial infarction, sepsis, and pneumonia. Patients with ESRD represent a sicker population at baseline, with careful consideration needed to determine surgical candidacy. In medically sick patients with comorbidities, medical management may be a reasonable option if delaying surgery is necessary.

**Table 2:** Literature review of tumoral calcinosis cases involving the cervical spine. Source: [8,12-44] ESRD, end-stage renal disease; CREST, calcinosis, Raynaud phenomenon, esophageal dysmotility, sclerodactyly, and telangiectasia; ROM, range of motion

Author and Year	Sex	Age	Location	Etiology	Symptoms	Treatment
Kokubun et al., 1996	F	68	C1-C2	Idiopathic	Neck pain	Laminectomy
Ohashi et al., 1996	F	12	Upper/mid cervical spine	Idiopathic	Torticollis	Resection
Mooney and Glazier, 1997	M	1.4	C1-C2	Idiopathic	Torticollis	Resection
Durant et al., 2001	M	78	C1-C2	Osteoarthritis	Not reported	Resection and fusion
Durant et al., 2001	F	70	C4-C5	Idiopathic	Not reported	Laminectomy
Matsukado et al., 2001	F	54	C2-C4	ESRD	Pain, weakness, and spastic gait	Laminectomy and laminoplasty
Teng et al., 2006	F	56	C3-C5	CREST syndrome	Radiculopathy	Laminectomy
Smucker et al., 2006	M	60	C2-C3	Scleroderma	Myelopathy and gait dysfunction	Corpectomy and fusion
Smucker et al., 2006	F	59	C4-C5	Scleroderma	Neck pain	Laminectomy and fusion
Smucker et al., 2006	F	73	C3-C5	Scleroderma	Neck pain and hand dysfunction	Discectomy and fusion
Miyakoshi et al., 2007	M	54	C3-C4	Idiopathic	Myelopathy	Laminectomy
Carlson et al., 2007	F	39	C4-C5	ESRD	Quadriparesis and monoplegia	Resection and medical management
Jackson et al., 2007	F	29	C6-T2	ESRD	Back pain, hand weakness, and tingling	Resection and fusion
Tuy et al., 2008	F	50	C2-C3	ESRD	Neck pain	Evacuation
Remy-Leroux et al., 2009	F	29	C6-T1	ESRD	Enlarging neck mass	Parathyroidectomy
Wong et al., 2013	F	77	C4-C5	Idiopathic	Neck pain, numbness, and weakness	Discectomy and fusion
Lebl and Girardi, 2013	F	63	Unknown levels	Scleroderma	Neck pain and limited ROM	Medical management
Chang et al., 2013	F	44	C1-C3	ESRD	Neck pain, limited ROM, and headache	Laminectomy and fusion
Sunder et al., 2013	F	50	C7-T2	ESRD	Pain and swelling	Parathyroidectomy
Al-Khudairi et al., 2015	F	62	C2-C4	Scleroderma	Weakness, altered sensation, and limited ROM	Laminectomy and fusion
Sasaki et al., 2015	F	90	C3-C5	Idiopathic	Numbness and weakness	Laminectomy
Sambataro et al., 2015	F	62	C4-C6	Scleroderma	Pain and limited ROM	Resection
Tan and Suresh, 2016	F	79	C3-C5	Scleroderma	Asymptomatic	Observation
Nguyen et al., 2016	F	57	C4-C5	Systemic sclerosis	Central cord syndrome	Laminectomy and fusion
Motegi et al., 2019	F	54	C3-C5	Scleroderma	Pain, weakness, and numbness	Resection
Fatehi et al., 2016	F	73	C2-C3	ESRD	Painful enlarging mass	Medical management
Guo et al., 2017	F	55	C4-C6	ESRD	Numbness, dull pain, and limited ROM	Hemilaminectomy
Faraj et al., 2017	F	66	C6-C7	CREST syndrome	Pain, numbness, and tingling	Laminectomy and fusion
Karschnia et al., 2018	F	58	C4-C5	Scleroderma	Dysesthesia, weakness, and spastic gait	Laminectomy and fusion
Logothetis et al., 2018	M	67	C3-C4	Systemic sclerosis	Pain	Medical management
Steward et al., 2019	F	4 months	C1-C2	Idiopathic	Decreased ROM and hypotonia	Observation
Chan et al., 2020	F	54	C7-T1	Mineral oil injection and ESRD	Weakness and pain	Laminectomy
Kendal et al., 2020	M	65	C5-C6	ESRD	Neck pain and lower extremity weakness	Laminectomy and medical management
Wang et al., 2022	F	73	C1-C2	Hyperparathyroidism	Pain, dysphagia, and odontoid fracture	Laminectomy and fusion
Pham et al., 2022	F	62	C2-C5 and C7-T1	Systemic sclerosis	Spinal cord injury	Laminectomy
Inomata et al., 2023	F	57	C4-C5	Systemic sclerosis	Numbness, weakness, and gait disturbance	Discectomy and fusion
Current case, 2026	M	46	C2-C6	ESRD	Weakness	Laminectomy and fusion

Uremic tumoral calcinosis is a rare complication of renal failure, affecting 0.5%-3% of patients with chronic kidney disease [32]. The most important factor contributing to the development of tumoral calcinosis is hyperphosphatemia, which is amplified by end-stage renal disease [11,32]. Patients with tumoral calcinosis with continued metabolic disturbances have a recurrence rate of 33%; therefore, managing the underlying disease is critical [32]. The global incidence of chronic kidney disease in adults is 14.2%, with a rising prevalence [45]. By 2040, kidney disease is projected to be the fifth leading underlying cause of mortality globally [46]. As the number of patients with chronic kidney disease increases, the estimated incidence of tumoral calcinosis may rise and become a more commonly encountered pathology managed by spine surgeons [45].

## Conclusions

We present a case of cervical tumoral calcinosis secondary to uremia in a medically unstable 46-year-old man with chronic kidney disease. Although the spine is a rare location for tumoral calcinosis, it should be considered as a differential diagnosis in patients with underlying conditions such as ESRD. It is important to consider that these patients will be sicker at baseline, with the careful determination of surgical candidacy necessary based on comorbidities present. Due to the location and symptoms present in our patient, surgery was determined as the optimal treatment strategy. Through this case and a review of the literature, we demonstrate that surgical resection is an appropriate management strategy to alleviate symptoms from mass effect, and further medical management may be necessary to correct underlying electrolyte abnormalities and prevent recurrence.
